# Concentrations, Source and Risk Assessment of Polycyclic Aromatic Hydrocarbons in Soils from Midway Atoll, North Pacific Ocean

**DOI:** 10.1371/journal.pone.0086441

**Published:** 2014-01-23

**Authors:** Yuyi Yang, Lee Ann Woodward, Qing X. Li, Jun Wang

**Affiliations:** 1 Key Laboratory of Aquatic Botany and Watershed Ecology, Wuhan Botanical Garden, Chinese Academy of Sciences, Wuhan, Hubei Province, China; 2 U.S. Fish and Wildlife Service, Pacific Reefs NWRC, Honolulu, Hawaii, United States of America; 3 Department of Molecular Biosciences and Bioengineering, University of Hawaii at Manoa, Honolulu, Hawaii, United States of America; NIEHS/NIH, United States of America

## Abstract

This study was designed to determine concentrations of polycyclic aromatic hydrocarbons (PAHs) in soil samples collected from Midway Atoll and evaluate their potential risks to human health. The total concentrations of 16 PAHs ranged from 3.55 to 3200 µg kg^−1^ with a mean concentration of 198 µg kg^−1^. Higher molecular weight PAHs (4–6 ring PAHs) dominated the PAH profiles, accounting for 83.3% of total PAH mass. PAH diagnostic ratio analysis indicated that primary sources of PAHs in Midway Atoll could be combustion. The benzo[a]pyrene equivalent concentration (BaP_eq_) in most of the study area (86.5%) was less than 40 µg kg^−1^ BaP_eq_ and total incremental lifetime cancer risks of PAHs ranged from 1.00×10^−10^ to 9.20×10^−6^ with a median value of 1.24×10^−7^, indicating a minor carcinogenic risk of PAHs in Midway Atoll.

## Introduction

Polycyclic aromatic hydrocarbons (PAHs) are an important group of environmental pollutants. They are introduced into the environment from both natural (e.g., oil seeps, forest fires and volcanic activity) and anthropogenic sources (e.g., petrochemical industrial effluents, coal tar processing wastes, combustion processes) [1]–[3]. PAHs may accumulate in the organisms due to their low solubility and high octanol-water partition coefficient and undergo long-range transport [4]–[6]. Furthermore, PAHs present potential carcinogenic risks to residents [7]. Thus, 16 PAHs are selected as the priority pollutants due to their frequency and/or risk by the U.S. Environmental Protection Agency [8].

Soil is the primary steady reservoir and sinks for PAHs in the terrestrial environment, because PAHs are readily absorbed by organic matter in soil and difficult to degrade [9]. Furthermore, the accumulation of PAHs in soil may lead to contamination of food chains, which could cause a potential risk to human health [10], [11]. Therefore, concentrations of PAHs in soil have been widely investigated in urban, rural, industrial and agricultural areas of mainland [1], [12], [13]. However, less data on concentrations of PAHs in soils of atolls and islands have been reported. Such data are required for understanding the potential risk to biota inhabiting the island and global distribution of PAHs.

Midway Atoll is located in the North Pacific Ocean, approximately 1100 miles northwest of Oahu, Hawaii. The atoll is comprised of two main islands, Sand and Eastern, and one smaller islet, enclosed within a reef approximately 8 km long. It is the home to a variety of seabirds, Hawaiian green sea turtles, Hawaiian monk seals, and spinner dolphins. It played a historical role in World War II and was altered very heavily by the military during the war and afterwards. This study intends to be a comprehensive study on PAHs in soils of Midway Atoll. The main objectives were: (1) to determine concentrations and compositions of PAHs; (2) to elucidate potential sources by PAHs diagnostic ratio analysis; and (3) to evaluate the possible carcinogenic risk of PAHs in the soil of Midway Atoll.

## Materials and Methods

### Study Area and Sample Collection

**Figure 1:**
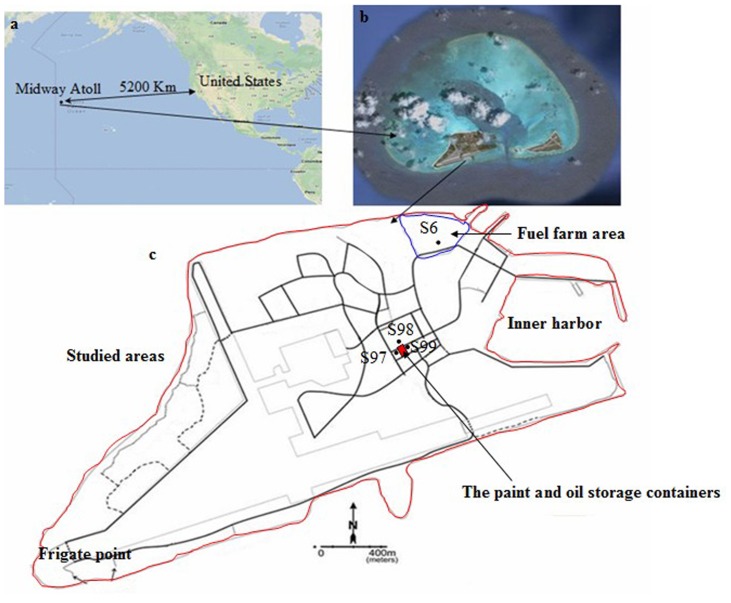
Locations of study area on Midway Atoll, the Pacific Ocean (a: The location of Midway Atoll in the Pacific Ocean; b: The Sand Island and Eastern Island of Midway Atoll; c: The main island of Midway Atoll (Sand Island) was the studied areas (Red line); Red area in the figure: The paint and oil storage containers; Blue line in the figure: Fuel farm area).

Midway Atoll is located at the northwest end of the Hawaiian Islands archipelago, at 28.208 °N latitude and −177.379°W longitude (Fig. 1). Midway Atoll had the land area of 5 km^2^ with northwest monsoon in winter. Soil has been augmented on Sand Island using naturally occurring guano from seabirds, as well as a shipment of 9,000 tons of soil in the early 1900’s from Oahu and Guam. The latter soil augmentation was done to facilitate growing vegetables on the island, and to extend the runway. The main textural class in Midway Atoll is sandy soil. One hundred and eleven samples of surface layers of soil (0–15 cm) from Midway Atoll by grid sampling strategies were collected in June 2009. Samples were hand dug with a trowel that was cleaned off between samples. A minimum of 50 g of soil per sample was collected for analysis. All samples were lyophilized, ground to pass through a sieve of 2 mm openings, and stored in an amber glass container at −25°C. All samples were collected under the permit of the U.S. Fish and Wildlife Service.

### Sample Preparation, Extraction and Cleanup

To determine PAHs in soils, an amount of 5 g soil was extracted with a supercritical fluid extractor SFX 220 (Isco, Inc., Lincoln, NE) according to the procedure previously described [14]. The extract was dried with anhydrous sodium sulfate (3 g) and rinsed with hexane (3 ml). The concentrated extract in hexane was cleaned up through an 8 mm i.d. aluminum/silica column. The column was packed with neutral silica (4.0 g, 3% deactivated), neutral alumina (2.0 g, 6% deactivated) and anhydrous sodium sulfate (1 cm) from the bottom to the top [15]. The column was eluted with 20 ml of solvent mixture (methylene chloride/hexane 1∶1) to yield a fraction containing PAHs. The samples were concentrated to 20 µL under a gentle stream of high purity nitrogen.

### Analysis of PAHs

The samples were analyzed on a Varian Saturn 2000 (Palo Alto, CA) gas chromatograph with mass spectrometric (ion trap) detection (GC/ITMS). The PAHs were separated by a capillary column DB-5MS (J and W Scientific Inc., 30 m, 0.25 mm i.d., 0.25 µm film thickness). The oven temperature was started at 50°C for 3 min, increased to 200°C at a rate of 10°C min^−1^, and increased to 280°C at a rate of 5°C min^−1^ and held for 8 min. The injector temperature was set at 280°C. Helium was used as the carrier gas at a constant flow rate of 1 ml min^−1^. External calibration was done for each PAH using a certified mixture to determine 16 US-EPA priority PAHs.

### Quality Assurance and Quality Control (QA/QC)

Average PAH recoveries and relative standard deviation (RSDs) were first obtained to evaluate the method performance by multiple analyses of 10 soil samples spiked with PAH standard (Accustandard, New Haven, CT), which contained 16 priority PAHs. The 16 PAHs were naphthalene (Nap), acenaphthylene (Acy), acenaphthene (Ace), fluorene (Flr), phenanthrene (Phn), anthracene (Ant), fluoranthene (Fla), pyrene (Pyr), benz[*a*]anthracene (BaA), chrysene (Chy), benzo[*b*]fluoranthene (BbF), benzo[*k*]fluoranthene (BkF), benzo[*a*]pyrene (BaP), dibenz[*a,h*]anthracene (DibA), benzo[*ghi*]perylene (BghiP) and indeno[*1,2,3-cd*]pyrene (InP). The spike level of each PAH was approximately 50–500 µg kg^−1^. A solvent blank and matrix blank were analyzed through the entire procedure prior to and after every 10 samples. Standard solutions of PAHs were run at the beginning of sample analysis to determine the relative response factors and evaluate peak resolution. Each sample was analyzed in triplicate unless otherwise stated.

Limits of detection (LOD) were determined as signals 3 times the background signal. Peaks that were smaller than 3 times the signal-to-noise ratio were not considered. The LOD for PAHs ranged from 10 to 500 pg g^−1^. The average recoveries of PAHs were 85–115% for 10 soil samples varying with the physicochemical properties of individual PAH.

### PAH Diagnostic Ratios Analysis

PAH diagnostic ratios have recently come into common use as a tool for identifying and assessing pollution sources. These ratios distinguish PAH pollution originating from petroleum products, petroleum combustion and biomass or coal burning, such as Ant/(Phn+Ant), Fla/(Pyr+Fla), InP/(InP+BghiP) [16], [17]. The compounds involved in each ratio have the same molar mass, so it is assumed they have similar physicochemical properties. Based on the PAH isomer ratios in source identification compiled by Yunker et al [18], the Fla/(Fla+Pyr) ratio <0.4 indicates petroleum input as a source; 0.4–0.5 indicates petroleum (liquid fossil fuel, vehicle and crude oil); and >0.5 indicates combustion of biomass and coal. In addition, an Ant/(Phe+Ant) <0.1 implies a petroleum source, >0.1 implies combustion as a source [10], [18].

### Risk Assessment

Toxicity equivalent (TEQ) method was used to assess the ecotoxicological risk at a specific site. The total BaP equivalent concentration (BaP_eq_) was calculated by the sum of BaP_eq_ for each PAH using toxicity equivalent factors [19].

About 20 people live on Midway Island, but they form a complete and mutually interdependent community. Therefore, the incremental lifetime cancer risk (ILCR) was employed to evaluate the potential risk of PAHs in soils of Midway Atoll for human health in this study. The ILCRs for adults in terms of direct ingestion, dermal contact, and inhalation were calculated using the following equations [5]:
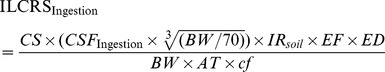
(1)

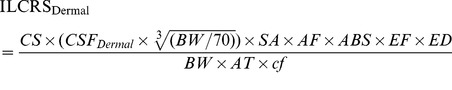
(2)

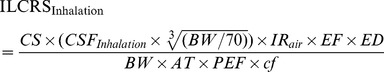
(3)where CS is the PAH concentration of soils (µg kg^−1^), which was obtained by converting concentrations of PAHs according to toxic equivalents of BaP using the toxic equivalency factor (TEF in Table 1) [20]. The carcinogenic slope factor (mg kg^−1^ day^−1^)^−1^ (CSF) was based on the cancer-causing ability of BaP: CSF_ingestion_, CSF_Dermal_ and CSF_Inhalation_ of BaP were 7.3, 25 and 3.85 (mg kg^−1^ day^−1^)^−1^, respectively [13]. BW is body weight (kg): 70 kg; AT is average life span (year): 70 years; EF is exposure frequency (days year^−1^): 350 days year^−1^; ED is the exposure duration (year): 30 years; IR_soil_ is the soil intake rate (kg day^−1^): 0.0001 kg day^−1^; IR_air_ is the inhalation rate (m^3^ day^−1^): 20 m^3^ day^−1^; SA is the dermal surface exposure (cm^2^ day^−1^): 5000 cm^2^ day^−1^; cf is the conversion factor: 10^6^; AF is the dermal adherence factor (kg cm^−2^): 0.00001 kg cm^−2^; ABS is the dermal adsorption fraction (unitless): 0.1; and PEF is the soil dust produce factor (m^3^ kg^−1^): 1.32×10^9^ m^3^ kg^−1^ [5], [8]. The total risks were the sum of risks of ILCRs in terms of direct ingestion, dermal contact, and inhalation.

**Table 1:** Concentrations of PAHs in soils collected from Midway Atoll (µg kg^−1^ dry weight).

PAHs	TEF	Minimum	Maximum	Mean	Median	Frequency	Type of PAHs	Soil guidelines
Nap	0.001	1.20	108	14.0	9.39	100%	LMW	5000
Acy	0.001	ND	10.0	0.61	0.05	60.4%	LMW	–
Ace	0.001	ND	5.30	0.40	0.09	60.4%	LMW	–
Flr	0.001	ND	6.83	0.84	0.66	88.3%	LMW	–
Phn	0.001	0.41	308	14.6	4.29	100%	LMW	5000
Ant	0.01	ND	41.6	2.61	0.41	66.7%	LMW	–
Fla	0.001	ND	649	35.4	3.57	99.1%	HMW	–
Pyr	0.001	ND	542	31.1	3.90	99.1%	HMW	10×10^3^
BaA	0.1	ND	308	17.5	2.09	91.9%	HMW	1000
Chy	0.01	ND	363	23.3	3.10	94.6%	HMW	–
BbF	0.1	ND	339	20.5	2.26	89.2%	HMW	1000
BkF	0.1	ND	150	8.68	1.11	85.6%	HMW	1000
BaP	1.0	ND	197	13.6	3.20	92.8%	HMW	1000
InP	0.1	ND	170	6.58	0.16	55.0%	HMW	1000
DibA	1.0	ND	30.2	1.85	0.00	45.0%	HMW	1000
BghiP	0.01	ND	169	6.59	1.23	65.7%	HMW	–
LMW PAHs		2.86	374	33.1	19.2			
HMW PAHs		ND	2830	165	21.1			
∑PAHs		3.55	3200	198	42.4			
LMW/HMW		0.05	16.9	1.52	0.62			
Total BaP_eq_		ND	324	21.2	4.38			
∑BaP_eq_ of 10 PAHs		ND	262	17.2	3.79			

LMW PAHs denote low molecular weight 2–3 ring PAHs; HMW PAHs denote high molecular weight 4–6 ring PAHs; TEF denotes toxic equivalency factor [33]; BaPeq denotes Bap equivalent concentration. ND: not detected.

Soil guidelines: guidelines for residential and parkland soil, NOAA-National Oceanic and Atmospheric Administration.

∑BaP_eq_ of 10 PAHs: Nap, Phn, Ant, Fla, Chy, BaA, BaP, BkF, InP, BghiP.

## Results and Discussion

### PAH Profiles in Soils of Midway Atoll

**Figure 2:**
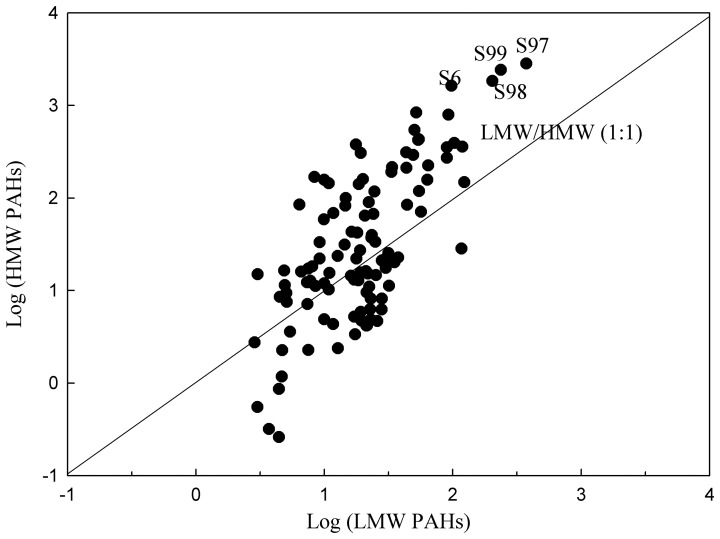
The logarithmic plot of LMW and HMW PAH concentrations of sampling sites on Midway Atoll.

Table 1 shows the descriptive statistics for concentrations of PAHs in soils from Midway Atoll. The overall concentration of 16 US EPA priority PAHs in surface soils ranged from 3.55 to 3200 µg kg^−1^ dry weight with a mean concentration of 198 µg kg^−1^. The detection frequencies of Nap and Phn were the highest (100%), followed by Fla (99.1%) and Pyr (99.1%). The detection frequency of DibA was the lowest among the 16 PAHs at a detection rate of 45.0%. The concentrations of lower molecular weight PAHs (LMW, i.e., 2–3 ring PAHs) in soils ranged from 2.86 to 374 µg kg^−1^ with a mean concentration of 33.1 µg kg^−1^. The concentrations of higher molecular weight PAHs (HMW, 4–6 ring PAHs) in soils ranged from ND (not detected) to 2830 µg kg^−1^ with a mean concentration of 165 µg kg^−1^. Most of the sampling sites (107 sites) had concentrations of LMW PAHs of <150 µg kg^−1^ and HMW PAHs of <1000 µg kg^−1^ (Fig. 2). Only 4 sites (S6, S97, S98 and S99) had concentrations of LMW PAHs of >150 µg kg^−1^ and HMW PAHs of >1500 µg kg^−1^. The high total PAH concentrations (S97, S98 and S99) were observed around the paint and oil storage on Midway Atoll. Site S6 having high total PAH concentrations located in fuel farm area (Fig. 1). Most of total PAH concentrations were distributed in the low concentration range with 50% of the samples showed concentrations less than 42.4 µg kg^−1^ (Median values in Table 1). The concentrations of individual PAH also showed a similar statistical characteristic for distribution, i.e., the median values were less than the average values. Compared with the established soil quality guidelines those from the National Oceanography and Atmospheric Administration (NOAA), concentrations of individual PAHs in soils of Midway Atoll were less than the guideline values (Table 1).

**Figure 3:**
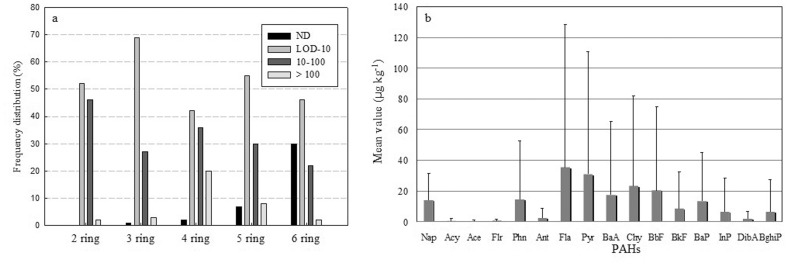
The frequency distribution of different ring PAHs (a) and mean concentrations of individual PAHs (b) on Midway Atoll.

Fig. 3a shows frequency distribution for the concentration of different PAHs analogs in all soil samples from Midway Atoll, indicating that most of soil samples had levels of different PAH analogs ranging from LOD to 10 µg kg^−1^. A two-ring PAH (Nap) was detected in all soil samples. The frequency distribution of three-rings PAHs in the range of LOD-10 µg kg^−1^ even reached 69%. It was notable that six-ring PAHs was not detected in 30% of the samples. However, 20% of the samples had levels of four-ring PAHs more than 100 µg kg^−1^. Fig. 3b shows that Fla, Pyr, Chy and BbF were found to be the main soil pollutants in Midway Atoll with mean value more than 20 µg kg^−1^, which were four-ring PAHs except BbF.

Soils and sediments were considered as the primary steady sinks for PAHs in the environment. Table 2 summarizes PAHs concentrations (µg kg^−1^ dry weight) in soils/sediments from islands and bays. Low contents of PAHs were found on the islands which were less disturbed by human activities, such as James Ross Island in Antarctica [21] and Admiralty Bay in King George Island [22]. LMW PAHs had a high prevalence in James Ross Island, indicating long-range atmospheric transport was the main source for PAHs contamination [21]. High content of PAHs were found in sediments and/or soils of the densely populated areas of islands, such as Coastal areas in the Shetland and Orkney Islands [23] and Island of Bermuda [24]. In this study, the concentrations of PAHs in Midway Atoll soils were found to be higher than those of James Ross Island, but lower than those of densely populated Bermuda. Midway Atoll had been used as military bases. Human activities in Midway Atoll may play an important role in PAHs contamination. Through the Baseline Realignment and Closure process, the US Navy undertook a cleanup operation to remove many environmental contaminants that resulted from 90 years of military operations [25]. Contaminants included polychlorinated biphenyls (PCBs), PAHs, petroleum hydrocarbons, asbestos, pesticides such as dichlorodiphenyltrichloroethane (DDT) and dichlorodiphenyldichloroethylene (DDE), and numerous metals. The results of this study suggest that several areas require continued monitoring for possible further remediation, such as S6, S97, S98 and S99.

**Table 2:** PAH concentrations (µg kg-1 dry weight) in soils/sediments from islands and bays.

Islands and bays	Soils/sediments	Number of PAHs	Range	Median/mean	Reference
Admiralty Bay, King George Island,Antarctica	Sediments	>16*	9.45–270	62.2	[22]
Coastal areas in the Shetland and OrkneyIslands, Britain	Sediments	**	LOD-22600	**	[23]
James ROSS Island, Antarctica	Soils	16	34–171	**	[21]
Vasilievsky Island, Russia	Soils	11	0.197–8.20	1.97	[34]
Potter Cove, South Shetland Islands, Antarctica	Sediments	25	36.5–1910	484/90.4	[35]
Island of Bermuda, Britain	Sediments	13	33.0–10200	1910/1070	[24]
Midway Atoll, USA	Soils	16	3.55–3200	198/42.4	This study

* 16 US EPA PAHs with alkyl-naphtalenes and methyl-phenanthrenes.

** No detail information.

### Potential Source of PAHs in Midway Atoll

The concentrations and patterns of PAHs in soils could reflect the source characteristics [26]. The ratios of LMW/HMW higher than 1 indicated the contaminations were mainly due to the petrogenic sources (hydrocarbon compounds associated with petroleum). On the other hand, the pyrogenic PAHs (hydrocarbon compounds associated with the combustion of petroleum, wood and coal) often showed to be at a LMW/HMW ratio less than 1.0 [27]. The LMW/HMW ratios in Midway Atoll soils ranged from 0.05 to 16.9 with a mean value 1.52. Among all 111 samples, 44 sites had a ratio greater than 1, indicating existence of petrogenic sources of PAHs (Fig. 2). Furthermore, recent pollution of petrogenic PAHs could occur at these 44 sites of predominance of low ring PAHs, because LMW PAHs were more biodegradable and less lipophilic than HMW PAHs. Similar results were also found for the PAHs in soils from Beijing, Tianjin and surrounding areas, North China [28]. Pyrogenic PAHs may be the main source at the other 67 sites with a ratio of LMW/HMW less than 1, such as S6, S97, S98 and S99 (Fig. 2).

**Figure 4:**
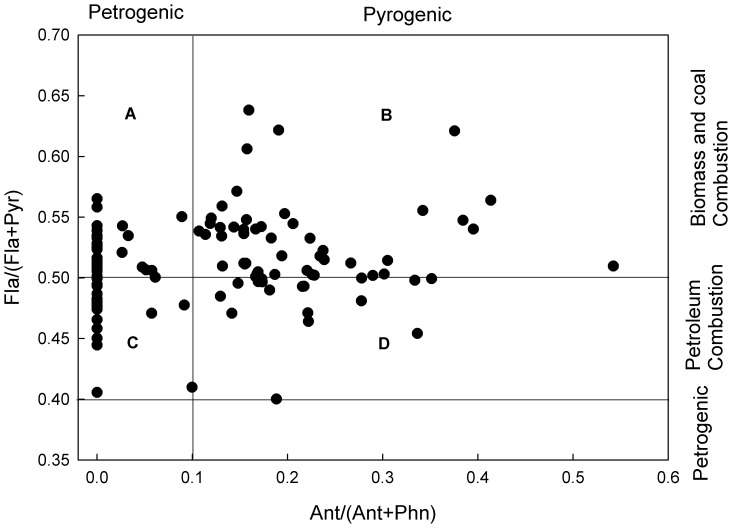
Cross plots for the ratios of Fla/(Pyr+Fla) and Ant/(Phn+Ant) (Fla: fluoranthene; Pyr: pyrene; Ant: anthracene; Phn:phenanthrene).

In the present study, the values of Fla/(Pyr+Fla) ranged from 0.40 to 0.64 and the values of Ant/(Phn+Ant) were between 0.0 to 0.54. Fig. 4 shows the cross plot of Fla/(Pyr+Fla) and Ant/(Phn+Ant), indicating that the sources of PAHs in soils could be classified into four distinct groups. About 15% and 45% of the sampling sites exhibited the typical characteristics of petroleum (liquid fossil fuel, vehicle and crude oil) combustion (Fig. 4D) and the signature of biomass and coal combustion (Fig. 4B), respectively. The remaining sites showed the signature of a mixture containinng petroleum and combustion (Fig. 4A and 4C). Hence, the primary source of PAHs in Midway Atoll could be considered as combustion. Midway Atoll is still an important military site and more than 90 years of military activities occurred on this island. The PAH sources of 55% of the sampling sites (Fig. 4A, 4C and 4D ) were related with petroleum, indicating petroleum played an important role for energy and military activities in this island. This result was different from James Ross Island, which was in Antarctic Peninsula and far from human activities. The LMW PAHs dominated the PAH contamination in James Ross Island, indicating the long-range atmospheric transport was the primary source [21].

### Risk Assessment of PAHs in Midway Atoll

Toxicity equivalent (TEQ) method was used to assess the ecotoxicological risk at a specific site. BaP_eq_ was calculated by the sum of BaP_eq_ for each PAH using toxicity equivalent factors [19]. In the present study, the total BaP_eq_ of 16 PAHs in soil samples were in the range of ND-324 µg kg^–1^ BaP_eq_ with a mean value of 21.2 µg kg^–1^ BaP_eq_ (Table 1). An half of all samples showed total BaP_eq_ concentrations less than 4.38 µg kg^–1^ BaP_eq_ and 86.5% of the samples had an exposure risk of less than 40 µg kg^–1^ BaP_eq_. According to the Canadian soil quality guidelines, soils containing <0.1 mg kg^–1^ BaP are considered uncontaminated, soils containing 0.1–1.0 mg kg^–1^ BaP are considered slightly contaminated and soils containing 1–10 mg kg^–1^ BaP are considered to be significantly contaminated [29]. In the present study, 95.5% of sample sites contained less than 0.1 mg kg^–1^ BaP_eq_ and only 5 sites had concentrations in the range 0.1–0.4 mg kg^–1^ BaP_eq_, indicating most of soils in Midway Atoll could be considered uncontaminated. The Dutch standards are environmental pollutant reference values (i.e., concentrations in an environmental medium) used in environmental remediation, investigation and cleanup. The target values for the various substances are related to a national background concentration that was determined for the Netherlands [30]. The total BaP_eq_ concentrations of 10 PAHs of the Dutch standards(i.e., Dutch target value) were in the range of ND-262 µg kg^–1^ BaP_eq_. Approximately one tenth of the sampling sites (11.3%) had values higher than the reference value 32.96 µg kg^–1^ BaP_eq_ [31], [32], indicating that 13 sampling soils of Midway Atoll had potential risk to human health.

Incremental lifetime cancer risk is a carcinogenic risk used to evaluate the human health risk. Generally, an ILCR between 10^−6^ and 10^−4^ indicates a potential risk [8]. Table 3 shows the ILCRs levels calculated in the Midway Atoll soils, indicating a low human health risk from the exposure of direct ingestion and inhalation. The ILCRs values of dermal contact with soils ranged from 9.52×10^−11^ to 8.69×10^−6^ with a mean value of 5.67×10^−7^ and 13.5% of the sampling sites exhibited ILCRs values of dermal contact exceeding 10^−6^, indicating a low potential carcinogenic risk via dermal contact at 15 sampling sites in Midway Atoll. The highest values of ILCRs was found in site S97, followed by S99, S98 and S6. This was accord with that highest concentrations of PAHs were found at these sites. Most of the total ILCRs were distributed in the low range and 86.5% of the samples showed the values less than 1.0×10^−6^, indicating a negligible carcinogenic risk of PAHs in Midway Atoll.

**Table 3:** Descriptive statistics of data on incremental lifetime cancer risks (ILCRs) in soils from Midway Atoll.

ILCRs	Minimum	Maximum	Mean	Median
Directingestion	5.56×10^−12^	5.07×10^−7^	3.31×10^−8^	6.85×10^−9^
Dermalcontact	9.52×10^−11^	8.69×10^−6^	5.67×10^−7^	1.17×10^−7^
Inhalation	4.33×10^−16^	4.05×10^−11^	2.65×10^−12^	5.47×10^−13^
Total ILCRs	1.00×10^−10^	9.20×10^−6^	6.00×10^−7^	1.24×10^−7^

## Conclusion

PAHs are widely distributed in the soils collected from Midway Atoll, in which HMW PAH concentrations ranged from ND to 2830 µg kg^–1^ with a mean concentration of 165 µg kg^–1^, accounting for 83.3% of the total PAH mass. The main PAH pollutants in Midway Atoll were found to be fluoranthene, pyrene, chrysene, benzo[b]fluoranthene and benzo[a]anthracene. Combustion of coal, petroleum and biomass was potentially the main source for PAH contamination in Midway Atoll. Majority of the sampling sites (95.5%) exhibited PAH concentrations less than 0.1 mg kg^–1^ BaP_eq_, which could be considered uncontaminated. The ILCRs of PAHs showed that PAH concentrations in most of the sampling areas in Midway Atoll are likely harmless to human health. However, the soil sites that contain an exhibiting carcinogenic risk still need management strategies.
